# Supplementary material for: Attitudes toward Aging among College Students: Results from an Intergenerational Reminiscence Project
Source: Behav Sci (Basel). 2023 Jun 28;13(7):538. doi: 10.3390/bs13070538 (PMC10376671; doi:10.3390/bs13070538)
Supplement: Supplementary file 1 [file behavsci-13-00538-s001.zip › behavsci-2449987-supplementary.pdf]

Supplemental Table S1. Characteristics of older adult participants at pretest

Characteristics	Sample (*N* = 61)		Sample (*N* = 103)	
	Mean (*SD*) or *n* (%)	Range	Mean (*SD*) or *n* (%)	Range
Age	75.15 (6.92)	52-91	74.92 (6.74)	65-91
AD8 Score	2.49 (1.16)	2-8	2.51 (1.07)	2-8
Number ADLs Help Needed	1.44 (1.44)	0-6	1.62 (1.69)	0-7
Number IADLS Help Needed	3.44 (2.19)	0-9	3.58 (2.27)	0-9
Gender				
women	50 (82.0%)		83 (80.6%)	
men	11 (18.0%)		20 (19.4%)	
Marital Status				
never married	7 (11.5%)		9 (8.7%)	
married/cohabitating	8 (13.1%)		16 (15.5%)	
widowed	26 (42.6%)		36 (35.5%)	
divorced	19 (31.1%)		37 (35.9%)	
separated	0 (0.0%)		2 (1.9%)	
other	1 (1.6%)		3 (2.9%)	
Education				
less than high school	5 (8.2%)		9 (8.7%)	
high school	25 (41.0%)		32 (31.3%)	
some college	15 (24.6%)		35 (34.0%)	
Bachelor’s degree	8 (13.1%)		13 (12.6%)	
graduate or professional degree	8 (13.1%)		14 (13.6%)	
Race/ethnicity				
white	30 (49.2%)		52 (50.5%)	
African American or Black	26 (42.6%)		44 (42.7%)	
Hispanic or Latino	2 (3.3%)		4 (3.9%)	
American Indian or Alaska Native	1 (1.6%)		1 (1.0%)	
multiple races or other	2 (3.3%)		2 (1.9%)	
Living Companion				

no one	42 (68.9%)	68 (66.0%)
spouse	6 (9.8%)	13 (12.6%)
adult child	6 (9.8%)	10 (9.7%)
sibling	2 (3.3%)	3 (2.9%)
other	5 (8.2%)	9 (8.7%)
Self-rated Health		
poor	5 (8.2%)	10 (9.7%)
fair	27 (44.3%)	44 (42.7%)
good	23 (37.7%)	40 (38.8%)
very good	5 (8.2%)	7 (6.8%)
excellent	1 (1.6%)	2 (1.9%)
Financial Strain		
not difficult at all	17 (27.9%)	23 (22.3%)
a little bit difficult	17 (27.9%)	27 (26.2%)
somewhat difficult	14 (23.0%)	29 (28.2%)
difficult	8 (13.1%)	16 (15.5%)
very difficult	5 (8.2%)	8 (7.8%)
Self-rated Stress		
extremely stressful	5 (8.3%)	10 (9.7%)
very stressful	4 (6.7%)	9 (8.7%)
moderately stressful	14 (23.3%)	24 (23.5%)
slightly stressful	20 (33.3%)	32 (31.4%)
not stressful	17 (28.3%)	27 (26.5%)
